# Application of Eight Machine Learning Algorithms in the Establishment of Infertility and Pregnancy Diagnostic Models: A Comprehensive Analysis of Amino Acid and Carnitine Metabolism

**DOI:** 10.3390/metabo14090492

**Published:** 2024-09-10

**Authors:** Rui Zhang, Lei Zhou, Xiaoyan Hao, Liu Yang, Li Ding, Ruiqing Xing, Juanjuan Hu, Fengjuan Wang, Xiaonan Zhai, Yuanbing Guo, Zheng Cai, Jiawei Gao, Jun Yang, Jiayun Liu

**Affiliations:** 1 Department of Clinical Laboratory Medicine, Xijing Hospital, Fourth Military Medical University, 127 Changle West Road, Xi’an 710033, China; ruizhang_zr@sina.com (R.Z.); iamwolf-snzq@163.com (L.Z.); a18332550530@163.com (X.H.); zhangzh@fmmu.edu.cn (L.Y.); pingfannvsheng@126.com (L.D.); xrqing210b@163.com (R.X.); 15349243701@163.com (J.H.); wfj1158007341@163.com (F.W.); xiaonanzhai_zxn@163.com (X.Z.); 15929568606@163.com (Y.G.); gjw941201@sina.com (J.G.); 2 Department of Clinical Labaratory, Xi’an People’s Hospital (Xi’an Fourth Hospital), Xi’an 710004, China; czh910419@163.com; 3 Comprehensive Cancer Center, Department of Entomology and Nematology, University of California, Davis, CA 95616, USA

**Keywords:** metabolomics, infertility, pregnancy, amino acids, carnitines

## Abstract

To explore the effects of altered amino acids (AAs) and the carnitine metabolism in non-pregnant women with infertility (NPWI), pregnant women without infertility (PWI) and infertility-treated pregnant women (ITPW) compared with non-pregnant women (NPW, control), and develop more efficient models for the diagnosis of infertility and pregnancy, 496 samples were evaluated for levels of 21 AAs and 55 carnitines using targeted high-performance liquid chromatography with tandem mass spectrometry (HPLC-MS/MS). Three methods were used to screen the biomarkers for modeling, with eight algorithms used to build and validate the model. The ROC, sensitivity, specificity, and accuracy of the infertility diagnosis training model were higher than 0.956, 82.89, 66.64, and 82.57%, respectively, whereas those of the validated model were higher than 0.896, 77.67, 69.72, and 83.38%, respectively. The ROC, sensitivity, specificity, and accuracy of the pregnancy diagnosis training model were >0.994, 96.23, 97.79, and 97.69%, respectively, whereas those of the validated model were >0.572, 96.39, 93.03, and 94.71%, respectively. Our findings indicate that pregnancy may alter the AA and carnitine metabolism in women with infertility to match the internal environment of PWI. The developed model demonstrated good performance and high sensitivity for facilitating infertility and pregnancy diagnosis.

## 1. Introduction

With an aging population and continuous decline in fertility rates, increasing the birth rate has emerged as a global issue that requires urgent attention. Infertility, estimated to affect 15% of couples worldwide, refers to the inability to conceive within one year of routine, unprotected sexual intercourse [1,2]. In addition to its physiological and psychological effects on women attempting to become pregnant, infertility is a risk factor for breast, endometrial, ovarian, and other cancers [3,4]. Infertility arises for various reasons, including endocrine, anatomical, genetic, and behavioral factors, obesity, ovulation disorders, and anatomical diseases of the pelvic organs [5,6]. 

Metabolomics provides a phenotypic fingerprint of a cell, tissue, or organism by measuring multiple metabolites directly [7,8]. Metabolomics has increasingly been employed for the identification of novel biomarkers for improved clinical strategies to uncover the causes and development of diseases [9]. Studies have demonstrated changes in glucose, amino acid (AA), lipid, steroid hormone, lactogen, and cortisol levels, and gut microbiota in the serum, urine, and tissues of infertile [10,11] and pregnant women [12,13]. However, the reasons for such changes remain unclear. While various studies on infertility and pregnancy have focused on the nutritional value of amino acids (AA) and carnitine, there is a considerable amount of literature reporting that amino acids and carnitine are also related to the pathogenesis of many other diseases [14,15]. Moreover, pregnancy has been shown to affect a woman’s immune system [16], suggesting that there may be other unknown functions of pregnancy that have not been discovered. However, studies on the metabolomic differences between pregnancies after fertility treatment and those without fertility treatment and the effects of pregnancy on metabolism-related aspects of infertility are limited, hindering our understanding of the underlying factors.

Patients with suspected infertility provide a detailed history and undergo a series of examinations, followed by laboratory assessments of ovulation, thyroid function, fallopian tube patency, and so on. Subsequently, infertility is diagnosed by combining the results of these and other multimodal assessments [17]. Infertility diagnosis is time-consuming and complicated by various factors [18]. Although imaging plays an indispensable role in assessing anatomical abnormalities, it is unsuitable for large-scale screenings [19]. Couples diagnosed with infertility could miss their window of opportunity for treatment, highlighting the importance of early intervention [20]. Therefore, a simple clinical screening index for the early prevention of and intervention in infertility is warranted.

Machine learning is a scientific discipline that focuses on how computers learn from data, including statistics and computer science [21]. The complexity and abundance of biological data present opportunities for analysis using machine learning techniques [22]. Although machine learning has been used in biology for decades, its application in medicine has attracted more and more attention in recent years [23], especially in the establishment of diagnostic models [24].

Thus, we aimed to investigate the metabolomic differences between NPWI, ITPW, PWI, and controls using targeted AA and carnitine metabolomics, as well as other clinical indicators. Diagnostic models were screened and validated using eight machine learning algorithms, providing new possibilities for the rapid diagnosis of infertility and pregnancy.

## 2. Materials and Methods

### 2.1. Participants

**Figure 1:**
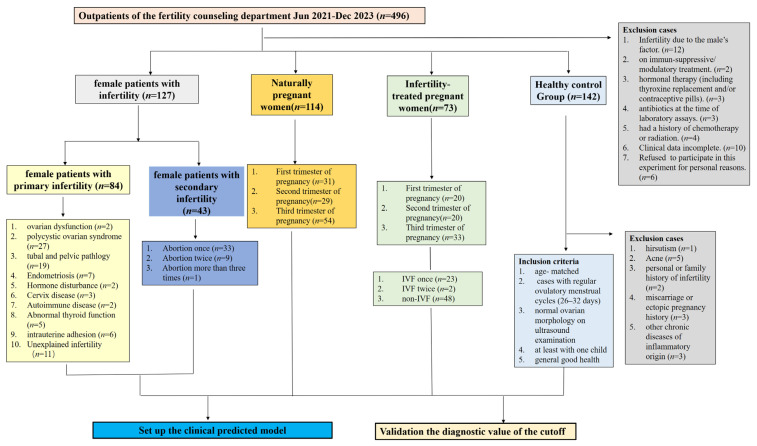
Patient information. Flowchart of inclusion and exclusion criteria for the female study groups.

Patients included in this study were enrolled from Fertility and Infertility Center of Xijing Hospital (Xi’an, China), from 1 June 2021, to 31 December 2023, and divided into four groups, NPWI (*n* = 127), ITPW (*n* = 73), PWI (*n* = 114), and NPW (controls; *n* = 128). Fertility was assessed and diagnosed by a specialist using revised American Society for Reproductive Medicine female infertility guidelines. All pregnancies were singleton, intrauterine pregnancies and matched for age and gestational week between groups. The controls were in generally good health, fertile with healthy ovarian function, had no adverse pregnancy history and other diseases, and had normal ovulatory cycles. The exclusion criteria across all four groups included the following: (1) history of immunosuppressive/modulatory treatment; (2) a history of hormonal therapy; (3) a history of taking antibiotic drugs; (4) a history of chemotherapy and radiation therapy; and (5) incomplete data or information (Figure 1).

The research protocol was approved by the Ethics Committee of Xijing Hospital (KY20212027-C-1). Written consent was obtained from all patients.

### 2.2. Sera Preparation

Blood samples were collected from the peripheral veins of all enrolled patients by a trained phlebotomist in the morning after an overnight fast. Venous blood was collected in accelerator separation gel, heparin anticoagulation, and ethylenediaminetetraacetic acid anticoagulant tubes for AAs, carnitine, biochemistry-related, immune-related, and routine blood tests.

### 2.3. Data Collection

Serum AA and carnitine levels were analyzed using high-performance liquid chromatography with tandem mass spectrometry (HPLC-MS/MS). Other clinical indicators were measured and statistically analyzed by the Clinical Laboratory Department of Xijing Hospital, which is accredited by the ISO15189 China National Laboratory Accreditation Committee (ISO 15189: 2022 Medical Laboratories—particular requirements for quality and competence. ISO, Geneva, Switzerland), with the results uploaded and maintained in the Laboratory Information System (LIS). Hospital records and patient information were obtained from the Hospital Information System and LIS. All data were tallied by at least three independent technicians and included patient demographics, physical and laboratory examinations, diagnoses, and other information (Figure 1 and Table S1).

### 2.4. MS Analysis

Detailed sample pre-treatment methods for HPLC-MS/MS are listed in the Supplemental Material. For the detection of the 21 AAs and 55 carnitines, 100% acetonitrile was used as the HPLC eluent. The optimized HPLC parameters are listed in Table S2. The sample injection volume was 20 μL. Two experiments were performed in one MS period in positive mode, with the optimized MS/MS parameters listed in Table S3. Experiment 1 scan type was neutral loss: loss of 102.00 Da starting from 140.00 Da and stopping at 280.00 Da; CEP starting from 13.07 and stopping at 17.46. Experiment 2 scan type was multiple reaction monitoring.

### 2.5. Statistical Analyses

MS data processing was performed using the Analyst 1.6.2 software (AB Sciex, Darmstadt, Germany). The ChemView software (version 1.6.1) was used to convert the MS results into quantitative data. Data were analyzed using SPSS software (version 23.0; IBM, Armonk, NY, USA). Normally distributed quantitative data were reported as the mean ± standard deviation and were compared between groups using one-way analysis of variance and least significant difference post-hoc test. All reported p-values were two-tailed, and the level of statistical significance was set at *p* < 0.05. GraphPad Prism version 5 (GraphPad Software, San Diego, CA, USA) and R version 3.6.2 (R Project for Statistical Computing) were used for data visualization. Orthogonal partial least squares discriminant analyses (OPLS−DA) were performed to identify differences between the groups. The odds ratios (OR) of the candidate indicators were evaluated and screened using binary logistic regression analysis. The diagnostic performance of the indicators was analyzed using receiver operating characteristic (ROC) curves. A Venn diagram was used to represent the indicators of consistent and differential expression. The biological functions and pathways of the differentially expressed metabolites were analyzed using the Small Molecule Pathway Database (SMPDB) and Kyoto Encyclopedia of Genes and Genomes (KEGG). Fisher’s exact test was used to evaluate the enrichment of predefined pathways in metabolites related to different groups.

### 2.6. Establishment of Diagnostic Models

**Figure 2:**
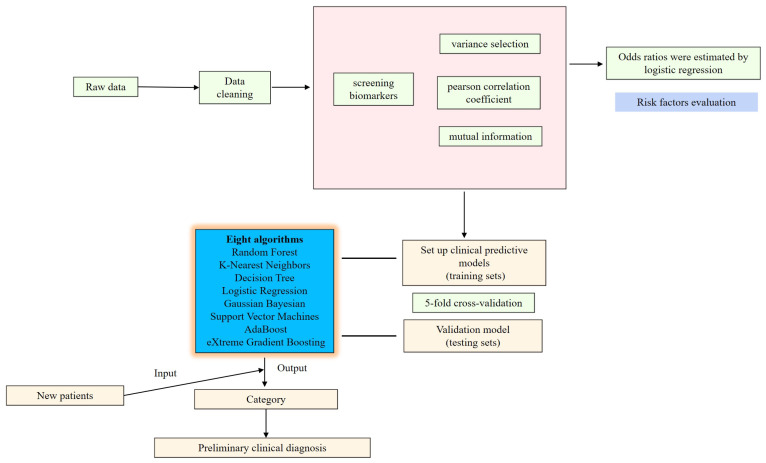
Flowchart of data processing.

To obtain newly developed biomarkers of infertility and pregnancy in women, we screened AAs, carnitines, and other clinical indicators using the following three methods: variance selection; Pearson correlation coefficient; and mutual information. Top 40 indicators from each method were intersected for the final selection of potential diagnostic indicators. Eight machine-learning classification algorithms were applied to establish diagnostic models to distinguish patients with infertility from healthy controls based on the selected indicators. The algorithms included random forest, K-nearest neighbors, decision tree, logistic regression, Gaussian Bayesian, support vector machines, AdaBoost, and extreme gradient boosting. To ensure the stability of the algorithm, it was cross validated with 5-fold of the data, with 4-fold used for training and 1-fold used for testing. Finally, the models were evaluated using sensitivity, specificity, accuracy, positive predictive value, negative predictive value, area under curve (AUC), and ROC curves. The flow chart is shown in Figure 2.

## 3. Results

### 3.1. Patient Characteristics

Figure 1 and Table S1 outline the main characteristics of the study participants and the analyte levels, respectively. The basic information of NPWI and control women is described in Section 2.1 and Figure 1. The two groups were matched for age and other information. ITPW were matched with PWI in terms of gestational age and weeks, with no statistical differences. The PWI comprised a first-, second-, and third-trimester group (mean age: 30.52 ± 3.41, 30.92 ± 5.22, and 30.50 ± 4.71 years, respectively; gestational ages: 11.04 ± 3.77, 19.00 ± 5.27, and 31.32 ± 2.96 weeks, respectively). The ITPW comprised a first-, second-, and third-trimester group (mean age: 31.21 ± 3.51, 31.37 ± 3.76, and 31.66 ± 3.52 years, respectively; gestational ages of 11.42 ± 2.39, 21.84 ± 4.83, and 31.78 ± 2.49 weeks, respectively).

NPWI showed significant differences in 96 indicators compared with control women. Among these indicators, 46 increased significantly and 50 decreased significantly (including AAs, carnitines, and their related ratios).

PWI showed significant differences in 106 indicators compared with healthy control women. Among them, 37 increased significantly and 69 decreased significantly (including AAs, carnitines, and their related ratios). The ITPW showed significant differences in 95 indicators when compared with those in control women. Among them, 35 increased significantly and 60 decreased significantly (including AAs, carnitines, and their related ratios). Conversely, only eight indicators in the ITPW were significantly different from those in the PWI. Among these eight, four were significantly higher and four significantly lower, with no significant differences in AAs, carnitines, or their related ratios.

### 3.2. Distribution of the Measured Indicators in Each Group

**Figure 3:**
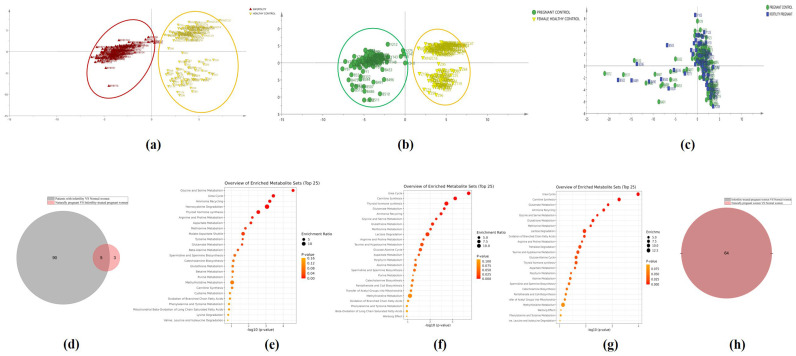
Multivariate data analysis. (**a**) Score plots of the supervised orthogonal partial least squares discriminant analyses (OPLS−DA) model. Control (yellow) and non-pregnant women with infertility (NPWI, red) groups. (**b**) Score plots of the supervised OPLS−DA model. Control (yellow) and pregnant women without infertility (PWI, green) groups. (**c**) Score plots of the supervised OPLS−DA model. infertility-treated pregnant women (ITPW, blue) and PWI (green) groups. (**d**) Venn diagram of the indicators (*p* < 0.05) between ITPW vs. PWI (pink) and NPWI vs. control women (gray). (**e**) Pathway analysis of significant metabolite changes between control group and NPWI group. (**f**) Pathway analysis of significant metabolite changes between control group and PWI group. (**g**) Pathway analysis of significant metabolite changes between control group and ITPW group. (**h**) Venn diagram of the pathways with significant changes between ITPW vs. control women and PWI (gray) vs. control women (pink).

The OPLS−DA model, employed to analyze overall differences in indicators between the groups, was statistically validated using permutation testing. The model revealed an excellent separation of samples between the NPWI and control women (R2Y = 0.362, Q2 = −0.364, Figure 3a) and between the PWI and control women (R2Y = 0.363, Q2 = −0.360, Figure 3b). No separation was observed between ITPW and PWI (Figure 3c). The Venn diagram of the indicators (*p* < 0.05) depicts a significant reduction in the indicator of difference when comparing ITPW vs. PWI to NPWI vs. control women, decreasing from 90 to 3 (Figure 3d).

### 3.3. Metabolic Pathways

Maternal physiological and metabolic processes are markedly affected during pregnancy [25]. Using enrichment and pathway analyses, the metabolic pathways corresponding to the differential metabolites (variable importance in the projection [VIP] > 1.0) were derived. According to the Human Metabolome Database, differential metabolites are involved in various metabolic pathways. The SMPDB differential pathway analysis comparing NPWI and control women, showed that the serine-glycine metabolism, the urea cycle, ammonia recycling, and other pathways were the most differentiated pathways (Figure 3e). The SMPDB pathway analysis comparing PWI and control women showed that the urea cycle and carnitine synthesis were the most differentiated pathways (Figure 3f). The SMPDB pathway analysis comparing ITPW and control women showed that the urea cycle and carnitine synthesis pathways were also the most differentiated (Figure 3g). Venn plots of differential metabolic pathways obtained by comparing PWI and ITPW with control women showed that the differential metabolic pathways were the same in the PWI and ITPW groups (Figure 3h). Similar results were obtained with KEGG pathway analysis, where 93.55% of the differential metabolic pathways were identical when comparing the PWI and ITPW groups with control women.

### 3.4. Performance Evaluation of Candidate Indices Using Classification Algorithm

**Figure 4:**
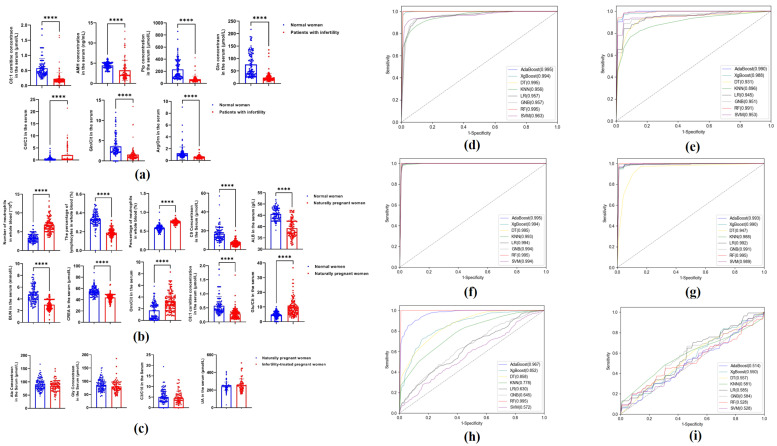
(**a**) C8:1, AMH, Pip, Gln, C4/C3, Gln/Cit, and Arg/Orn levels in NPWI and control women. (**b**) NEUT#, LYMPH%, NEUT%, C0, ALB, BU, CRE, Orn/Cit, Glu/Cit, and C8:1 levels in PWI and control groups. (**c**) Ala, Gly, C3/C16, and UA levels in the PWI and ITPW groups. (**d**) Receiver operating characteristic (ROC) curves of the model obtained from the training set of the seven different indices selected for NPWI and control women. (**e**) ROC curves of the model obtained from the verified set of the seven different indices selected for NPWI and control women. (**f**) ROC curves of the model obtained from the training set of the 10 different indices selected for the PWI and control women. (**g**) ROC curves of the model obtained from the verified set of the 10 different indices selected for the PWI and control women. (**h**) ROC curves of the model obtained from the training set of the four different indices selected for the PWI and ITPW. (**i**) ROC curves of the model obtained from the verified set of the four different indices selected for the PWI and ITPW. Abbreviations: C8:1, octenoyl carnitine; AMH, anti-Müllerian hormone; Pip, piperidine, Gln, glutamine; C4, butyryl carnitine; C3, propionyl carnitine; Cit, citrulline; Arg, arginine, Orn, ornithine; NEUT#, number of neutrophils; LYMPH%, lymphocyte percentage; NEUT%, neutrophil percentage; C0, free carnitine; ALB, albumin; BU, urea; CRE, creatinine; Glu, glutamic acid; Ala, alanine; Gly, glycine; C16, hexadecanoyl carnitine; UA, uric acid. ****, *p* < 0.0001.

Potential biomarkers for NPWI and PWI were cross screened based on differences in AAs, carnitine, and other clinical indicator expression levels, using three methods. Seven indicators [octenoyl carnitine (C8:1), anti-Müllerian hormone (AMH), Pip, Gln, C4/C3, Gln/Cit, and arginine/ornithine (Arg/Orn)] were selected as biomarkers of NPWI, with all being significantly lower (*p* < 0.0001) than in control women (Figure 4a). Ten indicators (number of neutrophils, lymphocyte percentage, neutrophil percentage, C0, albumin, urea (BU), creatinine, Orn/Cit, Glu/Cit, and C8:1) were selected as biomarkers for PWI, with all being significantly lower (*p* < 0.0001) than those in control women (Figure 4b). Additionally, Ala, Gly, C3/C16, and uric acid (UA) were selected to attempt to distinguish between PWI and ITPW; however, no significant differences were observed in the four indicators (Figure 4c), suggesting that they cannot be used to distinguish ITPW from PWI.

Eight classifier algorithms were implemented to guarantee the effectiveness of the diagnostic models established by candidate indicators. The ROC, sensitivity, specificity, and accuracy of the model obtained from the training set of the seven indices selected for the NPWI and control groups were >0.956, 82.89, 66.64, and 82.57, respectively (Table 1 and Figure 4d). The ROC, sensitivity, specificity, and accuracy of the model verified using the test set were >0.896, 77.67, 69.72, and 83.38, respectively (Table 1 and Figure 4e). For PWI and control women, the ROC, sensitivity, specificity, and accuracy of the model obtained using the 10 specific indicators in the training set were >0.994, 96.23, 97.79, and 97.69%, respectively (Table 2 and Figure 4f). The ROC, sensitivity, specificity, and accuracy of the model verified using the test set were >0.572, 96.39, 93.03, and 94.71, respectively (Table 2 and Figure 4g). For PWI and ITPW, the ROC, sensitivity, specificity, and accuracy of the model obtained in the training set of the four different indices were >0.956, 82.28, 7.41, and 62.37%, respectively (Table 3 and Figure 4h). However, the ROC, sensitivity, specificity, and accuracy of the model verified using the test set were >0.514, 64.87, 4.03, and 31.84, respectively (Table 3 and Figure 4i). The modeling of these four indicators cannot be used to distinguish between PWI and ITPW.

### 3.5. Selected Indices as Independent Predictors of Infertility in Women

To further explore the potential value of the candidate infertility biomarkers, binary logistic regression modeling was performed to determine whether the seven indicators predicted the occurrence of infertility in women (Table S4). The ratios of the occurrence of infertility in women for each one-unit increase in C8:1, AMH, and Arg/Orn were 50.610 (95% confidence interval [CI]: 2.601–984.879; *p* = 0.010), 1.522 (95% CI: 1.191–1.945; *p* = 0.001), and 128.985 (95% CI: 12.963–1283.447; *p* = 0.000), respectively. Additionally, the OR for each unit increase in C4/C3 was 0.680 (95% CI: 0.495–0.954; *p* = 0.025).

## 4. Discussion

Many couples struggle with infertility, with the risk of infertility reportedly equal among males and females [26,27]. The causes of infertility include genetic, hormonal, and environmental factors [28,29]. However, the cause of infertility remains unknown in ca. 30% of couples [30]. Extensive clinical work and research has been conducted on the treatment and diagnosis of infertility; however, additional research is necessary.

During pregnancy, the maternal body undergoes a series of changes, including changes in metabolism and immunity [31]. For example, large granular lymphocytes are reduced significantly during late pregnancy [32], concentrations of biochemical analytes are altered [33], activity of the complement system increases [34], and coagulation parameters and micronutrient concentrations change [35,36]. In the present study, the differences in AA and carnitine levels were more significant in PWI than in the control, consistent with previous reports [37]. However, the mechanisms and roles of altered AA and carnitine metabolism during pregnancy remain unclear. C0, Orn/Cit, Glu/Cit, and C8:1 were the indicators we screened that were significantly associated with pregnancy outcomes and significantly different from normal controls. Manta-Vogli PD et al. found that carnitine is abnormally active in the intermediate metabolism of pregnant women and newborns, and the conclusion that intake of long chain polyunsaturated fatty acids during pregnancy plays a beneficial physiological and metabolic role in the health of offspring [38] also demonstrates the importance of our screening indicators in pregnancy.

Our study also found that AA and carnitine metabolism were altered significantly in infertility. Cross-analysis with three screened methods and binary logistic regression modeling analysis revealed C8:1, Arg/Orn, and AMH with the greatest differences in the NPWI group that were also high-risk factors for disease, C4/C3 was low-risk factors for disease. In summary, AA and carnitine metabolism disorders play crucial roles in infertility development. However, few studies have reported on the differences in the AA and carnitine metabolism between PWI and ITPW, and on the effects of altered AA and carnitine metabolism during pregnancy in women with infertility. This study showed that the metabolic changes in ITPW were not significantly different from those in PWI. The proteomic results also showed a significant reduction in the indicator of difference when comparing polycystic ovary syndrome (PCOS) vs. control [39] with PCOS pregnancies vs. PWI [40], decreasing from 126 to 35, which also validates our conclusions. All these results suggest that gestation may remodel the AA and carnitine metabolism of women with infertility, thereby correcting the internal environment of women with infertility to that of PWI. In our study, we found that C8:1 is not only significantly correlated with both pregnancy outcome and infertility outcome, but also an indicator of significant difference compared with normal controls, indicating the importance of C8:1, but its role and application in the pathogenesis and treatment of infertility still need to be further investigated.

Monitoring hormonal changes during menstruation and ovulation is complex and not an integral part of routine care [41]. Clinical treatment is initiated only after several unsuccessful attempts. By that time, many couples may pass their optimal childbearing age [42]. Consequently, fertility treatments may become ineffective. The construction of a simple, convenient, and efficient prediction model that can be implemented in daily clinical practice remains necessary and clinically challenging. Junovich et al. reported that endometrial CD16^+^ natural killer (NK) cells, interleukin-6, and vascular endothelial growth factor were good parameters for the diagnosis of unexplained infertility [43], whereas He et al. reported that NK cells might be a potential predictor of women with PCOS (AUC = 0.69) [44]. However, the sensitivity, accuracy, convenience, and speed of infertility diagnosis can still be improved. The diagnostic model in this study was effective for the diagnosis of female infertility and pregnancy. We believe that the sensitivity and specificity of our model are significantly higher than those of conventional biochemical parameters and most reported parameters.

This study had several limitations. First, only a few samples from a single hospital were included, which may introduce a certain bias. Second, all the pregnant women in our study had a gestational age >8 weeks because it takes at least 4–5 weeks to confirm pregnancy, and pregnant women who go to the hospital for pregnancy tests are usually at more than five weeks of gestation. Therefore, including pregnant women whose gestational age is <8 weeks is difficult. This requires further studies to determine whether there are trends. In the follow-up study, we will recruit more pregnant women including all gestational weeks from multiple centers to further verify the effectiveness of our model.

## 5. Conclusions

In this study, targeted metabolomics was used to analyze the concentrations of 22 AAs, 55 carnitines, and their differences in the NPWI, PWI, ITPW, and NPW groups. The findings suggest that the gestational process may remodel the AA and carnitine metabolism in women with infertility, thereby correcting the internal environment of women with infertility to that of PWI. Moreover, relatively simple models were established with good performance and high sensitivity that may facilitate the early detection of infertility, enabling timely diagnosis and treatment within the optimal reproductive window.

## Figures and Tables

**Table 1:** Evaluation of the model for NPWI and control.

Algorithms		Sensitivity	Specificity	Accuracy	PPV	NPV	MCC	AUC
AdaBoost	Training set	100.00%	100.00%	100.00%	100.00%	100.00%	100.00%	0.995 (0.986–1.000)
	Test set	97.60%	97.45%	97.51%	97.58%	97.45%	95.03%	0.990 (0.978–1.000)
XGBoost	Training set	97.24%	99.33%	98.24%	99.41%	97.02%	96.50%	0.994 (0.984–1.000)
	Test set	92.16%	95.78%	93.76%	95.87%	91.82%	87.81%	0.988 (0.974–1.000)
DT	Training set	99.80%	99.77%	99.79%	99.81%	99.79%	99.58%	0.995 (0.986–1.000)
	Test set	91.61%	94.26%	92.52%	94.53%	91.14%	85.76%	0.931 (0.898–0.964)
KNN	Training set	82.89%	93.85%	88.07%	93.79%	83.10%	76.81%	0.956 (0.930–0.982)
	Test set	77.67%	90.23%	83.43%	90.27%	78.16%	68.16%	0.896 (0.856–0.936)
LR	Training set	92.31%	89.67%	91.08%	90.90%	91.32%	82.10%	0.957 (0.931–0.983)
	Test set	90.31%	86.24%	87.97%	87.99%	88.50%	76.51%	0.945 (0.916–0.974)
GNB	Training set	96.86%	66.64%	82.57%	76.44%	94.95%	67.32%	0.957 (0.931–0.983)
	Test set	96.17%	69.72%	83.38%	77.79%	94.26%	68.87%	0.951 (0.923–0.979)
RF	Training set	100.00%	100.00%	100.00%	100.00%	100.00%	100.00%	0.995 (0.986–1.000)
	Test set	96.89%	94.93%	95.84%	95.27%	96.52%	91.80%	0.991 (0.979–1.000)
SVM	Training set	92.33%	94.29%	93.26%	94.78%	91.69%	86.54%	0.963 (0.939–0.987)
	Test set	90.21%	94.94%	92.13%	95.02%	89.36%	84.76%	0.953 (0.926–0.980)

**Table 2:** Evaluation of the model for PWI and control.

Algorithms		Sensitivity	Specificity	Accuracy	PPV	NPV	MCC	AUC
AdaBoost	Training set	100.00%	100.00%	100.00%	100.00%	100.00%	100.00%	0.995 (0.986–1.000)
	Test set	99.13%	97.21%	98.23%	97.62%	99.13%	96.54%	0.993 (0.982–1.000)
XGBoost	Training set	98.88%	99.14%	99.01%	99.10%	98.92%	98.02%	0.994 (0.984–1.000)
	Test set	96.44%	95.50%	96.02%	95.71%	96.73%	92.19%	0.990 (0.977–1.000)
DT	Training set	100.00%	100.00%	100.00%	100.00%	100.00%	100.00%	0.995 (0.986–1.000)
	Test set	96.65%	93.03%	94.71%	93.46%	96.49%	89.81%	0.947 (0.917–0.977)
KNN	Training set	96.23%	99.12%	97.69%	99.09%	96.39%	95.41%	0.993 (0.982–1.000)
	Test set	94.44%	98.22%	96.44%	98.14%	95.11%	92.96%	0.988 (0.974–1.000)
LR	Training set	99.12%	97.79%	98.46%	97.83%	99.11%	96.92%	0.994 (0.984–1.000)
	Test set	99.13%	97.21%	98.23%	97.62%	99.13%	96.54%	0.992 (0.980–1.000)
GNB	Training set	98.24%	98.45%	98.35%	98.46%	98.25%	96.70%	0.994 (0.984–1.000)
	Test set	96.39%	98.08%	97.34%	98.42%	96.83%	94.86%	0.991 (0.979–1.000)
RF	Training set	100.00%	100.00%	100.00%	100.00%	100.00%	100.00%	0.995 (0.986–1.000)
	Test set	96.44%	97.21%	96.91%	97.62%	96.83%	94.05%	0.995 (0.986–1.000)
SVM	Training set	99.77%	99.34%	99.56%	99.34%	99.79%	99.12%	0.994 (0.984–1.000)
	Test set	98.26%	96.55%	97.33%	96.47%	98.26%	98.26%	0.989 (0.975–1.000)

**Table 3:** Evaluation of the model for PWI and ITPW.

Algorithms		Sensitivity	Specificity	Accuracy	PPV	NPV	MCC	AUC
AdaBoost	Training set	94.02%	84.26%	90.19%	90.23%	90.11%	79.30%	0.967 (0.943–0.991)
	Test set	64.87%	33.02%	52.15%	60.10%	37.78%	−2.12%	0.514 (0.429–0.599)
XGBoost	Training set	90.69%	56.89%	77.43%	76.52%	79.83%	51.76%	0.852 (0.799–0.905)
	Test set	77.04%	33.52%	59.72%	64.34%	47.89%	11.36%	0.550 (0.466–0.634)
DT	Training set	87.88%	67.64%	79.84%	81.84%	81.86%	59.15%	0.858 (0.806–0.910)
	Test set	75.69%	31.84%	31.84%	62.97%	49.81%	9.68%	0.557 (0.473–0.641)
KNN	Training set	82.28%	57.56%	72.58%	75.02%	67.89%	41.34%	0.778 (0.713–0.843)
	Test set	71.89%	37.88%	58.07%	64.63%	45.40%	9.83%	0.581 (0.498–0.664)
LR	Training set	87.80%	22.93%	62.37%	63.81%	55.33%	14.30%	0.630 (0.550–0.710)
	Test set	87.56%	20.51%	61.34%	62.90%	58.74%	13.17%	0.585 (0.502–0.668)
GNB	Training set	88.06%	28.44%	64.65%	65.61%	60.33%	20.65%	0.645 (0.566–0.724)
	Test set	87.39%	25.69%	63.49%	64.76%	55.16%	16.07%	0.584 (0.501–0.667)
RF	Training set	100.00%	100.00%	100.00%	100.00%	100.00%	100.00%	0.995 (0.986–1.000)
	Test set	72.01%	23.84%	52.67%	58.97%	38.51%	−3.44%	0.528 (0.443–0.613)
SVM	Training set	99.10%	7.41%	63.17%	62.42%			0.572 (0.489–0.655)
	Test set	96.59%	4.03%	60.23%	60.97%			0.528 (0.443–0.613)

## Data Availability

Data will be shared with other researchers for further analysis.

## Supplementary Materials

The following supporting information can be downloaded at: https://www.mdpi.com/article/10.3390/metabo14090492/s1, Table S1: List of indicator changes in serum. Table S2: High-performance liquid chromatography (HPLC) condition for amino acids and carnitines detection. Table S3: Optimized parameters for amino acids and carnitines detection using quadrupole-trap mass spectrometry. Table S4: Logistic regression analysis of predictive factors for NPWI.
