# Supplementary material for: Application of Eight Machine Learning Algorithms in the Establishment of Infertility and Pregnancy Diagnostic Models: A Comprehensive Analysis of Amino Acid and Carnitine Metabolism
Source: Metabolites. 2024 Sep 10;14(9):492. doi: 10.3390/metabo14090492 (PMC11433856; doi:10.3390/metabo14090492)
Supplement: Supplementary file 1 [file metabolites-14-00492-s001.zip › metabolites-3162722-supplementary.pdf]

Supplemental Material

Sample preparation for high-performance liquid chromatography with tandem mass spectrometry (HPLC-MS/MS)

For the analysis of AAs, alanine (Ala), arginine (Arg), aspartic acid (Asp), citrulline (Cit), glutamine (Gln), glutamic acid (Glu), glycine (Gly), homocysteine (Hcy), histidine (His), leucine (Leu), lysine (Lys), methionine (Met), ornithine (Orn), phenylalanine (Phe), piperidine (Pip), proline (Pro), serine (Ser), threonine (Thr), tryptophan (Trp), tyrosine (Try), and valine (Val) were labeled and extracted using the extraction reagent comprising internal standard solution $^{15}N^{13}C$-Gly, d_4-Ala, d_4C^{13}-Arg, d_3-Asp, d_2-Cit, d_3-Glu, d_3-Leu, d_3-Met, d_2-Orn, $^{13}C_6$-Phe, $^{13}C_6$-Tyr, and d_8-Val (Cambridge Isotope Laboratories). Free carnitine (C0), acetyl carnitine (C2), propionyl carnitine (C3), butyryl carnitine (C4), isovaleryl carnitine (C5), hexanoyl carnitine (C6), heptanoyl carnitine (C7), octanoyl carnitine (C8), nonanoyl carnitine (C9), decanoyl carnitine (C10), dodecanoyl carnitine (C12), tetradecanoyl carnitine (C14), hexadecanoyl carnitine (C16), heptanoyl carnitine (C17), octadecanoyl carnitine (C18), eicosanoyl carnitine (C20), docosanoyl carnitine (C22), eicosanoyl carnitine (C24), eicosanoyl carnitine (C25), eicosanoyl carnitine (C26), methylcrotonyl carnitine (C5:1), octenoyl carnitine (C8:1), decenoyl carnitine (C10:1), decadienyl carnitine (C10:2), decatrienoyl carnitine (C10:3), dodecenoyl carnitine (C12:1), tetradecenoyl carnitine (C14:1), tetradecadienoyl carnitine (C14:2), hexadecenoyl carnitine (C16:1), octadecenoyl carnitine (C18:1), octadecadienoyl carnitine (C18:2), eicosatenoyl carnitine (C20:1), eicosadienoyl carnitine (C20:2), eicosatrienoyl carnitine (C20:3), malonyl carnitine (C3DC), methylmalonyl carnitine (C4DC), glutaryl carnitine (C5DC), hexanedioyl carnitine (C6DC), octanedioyl carnitine (C8DC), sebacodioyl carnitine (C10DC), dodecanedioyl carnitine (C12DC), tetradecanedioyl carnitine (C14DC), cetradecanedioyl carnitine (C14DC), cetyl carnitine (C18:2), cis-acyl carnitine (C18:1), cicosadienoyl carnitine (C20:2), cicosapentaenoyl carnitine (carbonyl carnitine, C16DC), octadecanediacyl carnitine (C18DC), eicosanediacyl carnitine (C20DC), hydroxybutyryl carnitine (C4-OH), hydroxyisovaleryl carnitine (C5-OH), hydroxyhexanoyl carnitine (C6-OH), hydroxydodecanoyl carnitine (C12-OH), hydroxytetradecanoyl carnitine (C14-OH), hydroxyhexadecenoyl carnitine (C16:1-OH), hydroxyhexadecanoyl carnitine (C16-OH), hydroxyoctadecenoyl carnitine (C18:1-OH), hydroxyoctadecanoyl carnitine (C18-OH), and hydroxyeicosanoyl carnitine (C20-OH) were labeled and extracted using the extraction reagent which comprised internal standard solution free carnitine internal standard (d_9-C0), acetylcarnitine internal standard (d_3-C2), propionylcarnitine internal standard (d_3-C3), butyrylcarnitine internal standard (d_3-C4), isovalerylcarnitine internal standard (d_9-C5), octanoylcarnitine internal standard (d_3-C8), tetradecanoylcarnitine internal standard (d_9-C14), and hexadecanoylcarnitine internal standard (d_3-C16). All analyses in this research were performed using an LC-MS/MS system equipped with an Agilent 1200 HPLC system (Agilent1200, Waldbronn, Germany) and API 3200 QTRAP MS/MS system (Sciex, Darmstadt, Germany). Deionized water was obtained using a Milli-Q water purification system (Millipore, Billerica, MA, USA).

To detect the selected 21 AAs and 55 carnitines, 50 μL of serum was dripped on a blank blood collection filter paper and penetrated completely, subsequently, 3.5 mm of the dry serum filtration paper was removed using a stick. Metabolites were then extracted using 100 μL extraction solution for 15 min and transferred for N_2 drying. N-butanol hydrochloride solution (60 μL, 3 mol/L; prepared in a volume ratio of n-butanol to acetyl chloride at 9:1) was then added to the drying sample for derivatization and incubated at 65 °C for 20 min. N_2 drying was used for the derivatization solution, and 100 μL of acetonitrile was then added as a detection solution. The mass spectra of all metabolites to be tested were analyzed using a series of mass spectrometers, and their concentrations were calculated.

Supplementary Table S1. List of Indicator Changes in Serum

Descriptives	Mean				*P*			
					PWI	PWI	ITPW	NPWI
	PWI	ITPW	NPWI	NPW	ITPW	NPW	NPW	NPW
Age	30.580±4.500	31.810±3.250	31.610±5.600	31.670±3.610	0.337	0.275	0.326	0.411
Week	23.190±9.590	22.740±8.910						
AG	1.360±0.210	1.380±0.210	1.550±0.220	1.610±0.170	0.663	0.000	0.000	0.070
ALB	39.100±3.939	38.136±3.808	46.441±3.561	45.909±2.621	0.070	0.000	0.000	0.317
ALP	82.49±46.888	77.514±37.888	68.478±17.822	62.324±19.397	0.315	0.000	0.002	0.214
ALT	13.940±7.588	16.586±16.935	20.261±16.026	14.675±5.886	0.155	0.652	0.291	0.002
AST	17.580±6.406	19.686±14.748	20.333±8.597	17.559±4.281	0.109	0.986	0.104	0.035
AST/ALT	1.392±0.410	1.443±0.546	1.197±0.396	1.323±0.446	0.464	0.274	0.085	0.069
BACT	1846.992±6944.122	822.974±1701.159	1913.312±8237.225	1359.291±5456.129	0.321	0.668	0.663	0.662
BASO#	0.028±0.015	0.028±0.015	0.033±0.018	0.029±0.015	0.722	0.769	0.534	0.132
BASO%	0.002±0.004	0.002±0.004	0.005±0.005	0.006±0.005	0.705	0.000	0.000	0.372
BU	2.860±0.835	3.079±1.025	4.513±1.370	4.799±1.356	0.241	0.000	0.000	0.102
CAST	0.314±0.265	0.331±0.329	0.434±0.517	0.074±0.187	0.758	0.000	0.000	0.000
CL	102.876±2.083	103.989±2.718	102.771±3.228	104.083±1.452	0.135	0.160	0.917	0.173
CO2	20.760±2.316	21.211±2.509	22.786±1.720	24.933±1.656	0.503	0.000	0.000	0.015
CRE	42.837±6.834	44.338±15.76	53.239±10.177	54.919±8.104	0.373	0.000	0.000	0.290
Ca	2.156±0.251	2.097±0.218	1.985±0.308	2.169±0.274	0.439	0.888	0.434	0.050
CysC	0.706±0.224	0.769±0.373	0.704±0.144	0.780±0.119	0.069	0.018	0.743	0.023
DBIL	2.695±1.786	2.781±1.877	3.764±1.66	3.853±1.063	0.726	0.000	0.000	0.717
EC	39.506±71.844	27.155±31.170	36.522±44.719	11.074±21.970	0.122	0.001	0.092	0.010
EO#	0.071±0.057	0.114±0.266	0.087±0.093	0.093±0.071	0.031	0.203	0.281	0.744
EO%	0.008±0.007	0.013±0.025	0.014±0.017	0.017±0.013	0.046	0.000	0.088	0.177
ERY	0.072±0.272	0.113±0.456	0.814±1.131	0.231±0.678	0.723	0.166	0.352	0.000
FT3	4.389±0.786	4.452±1.162	4.998±1.438	4.503±0.846	0.773	0.559	0.812	0.005
FT4	14.864±3.014	15.834±3.339	16.66±3.823	15.513±1.984	0.121	0.245	0.595	0.020
G	29.122±3.384	28.01±3.608	30.306±3.791	28.788±2.547	0.035	0.467	0.137	0.003
GGT	12.81±7.487	14.586±19.538	23.551±23.67	14.373±3.98	0.449	0.461	0.927	0.000
Glucose	3.342±1.85	3.194±2.104	4.868±0.973	4.724±0.752	0.554	0.000	0.000	0.539
HCT	0.361±0.035	0.356±0.036	0.407±0.038	0.404±0.05	0.391	0.000	0.000	0.606
HGB	118.079±12.597	116.697±13.321	132.391±14.432	132.027±12.536	0.495	0.000	0.000	0.852
HyCAST	0.191±0.172	0.23±0.229	0.325±0.410	0.031±0.082	0.327	0.000	0.000	0.000
IBIL	6.991±3.855	7.146±3.016	7.539±3.321	8.256±2.321	0.757	0.005	0.026	0.153
K	3.911±0.279	4.06±0.329	4.025±0.358	4.033±0.295	0.113	0.256	0.816	0.945
KET	0.295±0.773	0.278±0.738	0.059±0.238	0.005±0.048	0.847	0.000	0.002	0.536
LAP	158.067±78.486	153.471±80.705	50.125±13.72	42.211±4.569	0.682	0.000	0.000	0.668
LEU	0.771±1.04	0.588±0.819	0.451±0.808	0.128±0.453	0.149	0.000	0.000	0.010
LYMPH#	1.657±0.431	1.787±0.627	1.762±0.451	1.913±0.508	0.099	0.000	0.103	0.048
LYMPH%	0.185±0.042	0.204±0.059	0.291±0.077	0.335±0.074	0.062	0.000	0.000	0.000
MCH	30.003±2.368	30.344±2.497	29.554±2.35	29.796±2.009	0.339	0.502	0.117	0.482
MCHC	326.703±10.888	327.894±10.398	325.725±12.258	324.473±10.51	0.489	0.136	0.043	0.452
MCV	91.749±5.357	92.482±6.232	90.635±5.397	91.776±4.488	0.373	0.969	0.382	0.152
MONO#	0.554±0.180	0.569±0.184	0.410±0.152	0.372±0.119	0.534	0.000	0.000	0.102
MONO%	0.062±0.016	0.064±0.017	0.065±0.015	0.065±0.017	0.343	0.171	0.808	0.930
MPV	10.894±1.229	10.653±0.980	10.444±0.722	11.395±1.018	0.139	0.000	0.000	0.000
MUCUS	0.749±1.250	0.876±1.324	1.784±2.189	30.594±83.028	0.988	0.000	0.000	0.001
NEUT#	6.849±1.860	6.497±1.898	4.159±2.217	3.356±1.035	0.178	0.000	0.000	0.002

NEUT%	0.744±0.048	0.718±0.066	0.626±0.085	0.58±0.086	0.030	0.000	0.000	0.000
NIT	0.036±0.188	0.019±0.137	0.020±0.140	0.009±0.096	0.475	0.181	0.677	0.658
Na	137.476±1.598	138.153±2.881	139.707±2.088	142.325±1.915	0.288	0.000	0.000	0.002
NonSEC	4.296±5.219	4.473±4.221	3.379±3.182	0.865±1.034	0.789	0.000	0.000	0.002
P-LCR	0.321±0.097	0.302±0.079	0.285±0.060	0.368±0.101	0.166	0.000	0.000	0.000
PDW	13.177±3.051	12.530±2.368	12.029±1.577	14.186±2.646	0.112	0.004	0.000	0.000
PH	6.669±0.735	6.603±0.706	5.833±0.683	6.098±0.561	0.557	0.000	0.000	0.017
PLT	216.723±57.856	219.106±56.505	252.884±62.808	244.384±60.951	0.793	0.000	0.005	0.333
PRO	0.298±0.453	0.300±0.368	0.235±0.352	0.027±0.149	0.967	0.000	0.000	0.000
PathCAST	0.109±0.146	0.096±0.171	0.101±0.171	0.038±0.133	0.603	0.014	0.065	0.052
RBC	4.430±2.583	4.224±2.348	4.448±0.505	8.858±39.484	0.951	0.126	0.158	0.172
URBC	11.558±14.049	10.543±17.455	217.152±908.182	6.433±8.282	0.996	0.981	0.986	0.308
RDW	45.017±4.821	45.444±5.660	42.084±2.365	43.018±2.497	0.509	0.000	0.000	0.135
SquaEC	36.653±72.365	22.202±29.008	33.770±43.763	7.995±17.172	0.073	0.001	0.135	0.009
T3	2.348±0.505	5.369±19.389	1.809±0.494	1.712±0.393	0.040	0.625	0.011	0.933
T4	130.292±33.010	139.534±25.465	104.212±24.002	94.046±14.017	0.061	0.000	0.000	0.008
TBA	2.387±1.634	2.291±1.186	3.703±2.419	2.851±1.623	0.775	0.210	0.160	0.125
TBIL	9.686±5.219	9.927±4.414	11.303±4.649	12.109±3.279	0.730	0.000	0.002	0.249
TG	12.449±8.381	9.168±7.151	10.241±14.527	3.593±5.873	0.188	0.000	0.013	0.000
TP	68.220±5.245	66.211±5.497	76.733±5.309	74.697±3.766	0.008	0.000	0.000	0.007
TSH	1.980±1.050	1.709±1.065	2.647±1.606	2.570±1.224	0.298	0.012	0.001	0.710
UA	237.507±60.280	265.068±101.784	295.172±66.825	272.103±46.497	0.020	0.001	0.526	0.034
UBG	0.540±0.277	0.526±0.112	0.500±0.000	0.248±0.251	0.733	0.000	0.000	0.000
WBC	11.982±28.243	10.511±11.445	6.399±2.445	7.179±12.232	0.560	0.028	0.178	0.748
UWBC	40.887±68.009	59.557±70.474	32.647±64.853	9.800±11.432	0.328	0.167	0.051	0.294
WBCClumps	1.079±4.213	0.448±1.124	0.366±1.121	0.006±0.035	0.159	0.047	0.440	0.543
XTAL	4.325±8.517	1.100±0.000	64.817±116.295	2.700±3.536	0.120	1.000	0.183	1.000
aTG	35.296±70.730	80.133±172.563	161.086±636.67	44.912±91.721	0.675	0.920	0.757	0.179
m-AST	6.133±2.069	7.216±6.918	8.800±4.829	7.432±2.651	0.144	0.114	0.805	0.273
Ala	90.212±21.519	84.543±21.012	112.951±29.214	112.314±30.760	0.175	0.000	0.000	0.860
Arg	18.651±4.270	18.837±4.616	18.357±6.927	23.340±12.457	0.961	0.165	0.236	0.128
Asp	23.940±12.017	22.233±9.940	19.473±8.087	33.749±28.985	0.478	0.000	0.000	0.000
Cit119	10.546±5.256	8.659±4.342	18.429±9.525	17.810±11.195	0.141	0.000	0.000	0.576
Cys	0.287±0.453	0.294±0.196	0.257±0.759	0.427±0.227	0.935	0.085	0.134	0.028
Gln	32.311±32.127	30.591±28.420	23.458±17.509	77.056±49.784	0.718	0.000	0.000	0.000
Glu	80.659±22.097	75.889±21.103	99.432±31.854	81.801±29.528	0.230	0.746	0.137	0.000
Gly	86.745±21.515	80.208±23.340	171.481±198.888	119.303±45.864	0.673	0.018	0.012	0.000
Hcy	13.462±3.834	14.009±3.575	11.977±1.459	14.933±5.848	0.321	0.003	0.094	0.000
His	16.644±81.411	7.974±13.178	13.090±68.995	28.076±30.276	0.284	0.112	0.013	0.032
LeuIle	47.016±12.936	46.927±11.438	54.136±14.888	65.958±21.635	0.971	0.000	0.000	0.000
Lys	2.595±2.740	2.383±1.760	2.353±3.483	5.930±4.526	0.672	0.000	0.000	0.000
Met	12.138±12.517	11.205±4.230	12.381±19.154	22.320±21.444	0.682	0.000	0.000	0.000
Orn	29.099±12.122	26.901±11.016	35.276±10.324	26.668±18.280	0.265	0.166	0.906	0.000
Phe	29.841±7.788	28.756±7.310	31.429±9.137	31.050±10.967	0.434	0.327	0.099	0.751
Pip	94.511±91.099	87.817±84.505	69.830±51.271	233.567±158.053	0.644	0.000	0.000	0.000
Pro	243.134±86.852	228.921±82.296	424.528±189.544	278.989±111.840	0.486	0.048	0.014	0.000
Ser	21.082±8.530	20.769±6.203	16.502±10.832	24.496±8.162	0.809	0.003	0.004	0.000
Thr	17.736±8.654	17.375±7.603	12.096±10.706	23.524±14.699	0.815	0.000	0.000	0.000
Trp	14.041±4.992	14.396±4.821	17.163±7.074	21.085±13.390	0.768	0.000	0.000	0.000
Tyr	19.067±5.509	18.254±4.599	23.503±6.412	27.885±10.683	0.457	0.000	0.000	0.000
Val	81.369±182.202	64.481±26.219	76.754±172.599	117.551±56.136	0.368	0.030	0.005	0.012
Arg/Orn	0.717±0.247	0.794±0.355	0.548±0.216	1.224±1.081	0.637	0.001	0.009	0.000
Cit/Arg	0.599±0.359	0.491±0.330	1.015±0.368	1.159±1.337	0.303	0.000	0.000	0.112

Cit/Phe	0.372±0.208	0.323±0.205	0.892±2.802	0.666±0.616	0.818	0.126	0.112	0.225
Gln/Cit	3.630±3.306	3.439±2.144	1.568±1.623	3.620±2.577	0.596	0.975	0.615	0.000
Glu/Cit	9.558±5.556	9.914±4.900	6.753±4.276	4.448±2.236	0.584	0.000	0.000	0.000
Gly/Ala	0.990±0.258	0.981±0.280	1.726±2.777	1.103±0.400	0.964	0.548	0.563	0.001
Leu/Ala	0.539±0.196	0.574±0.159	0.488±0.118	0.624±0.303	0.221	0.001	0.088	0.000
Leu/Phe	1.617±0.448	1.696±0.525	1.779±0.424	2.272±0.871	0.345	0.000	0.000	0.000
Met/Leu	0.267±0.312	0.240±0.064	0.226±0.256	0.376±0.452	0.543	0.005	0.002	0.000
Met/Phe	0.417±0.388	0.411±0.225	0.407±0.528	0.886±1.081	0.947	0.000	0.000	0.000
Orn/Cit	3.231±1.534	3.368±1.471	2.344±1.243	1.703±1.411	0.519	0.000	0.000	0.000
Phe/Tyr	1.613±0.344	1.613±0.337	1.347±0.263	1.208±0.456	0.989	0.000	0.000	0.003
Thr/Cit	2.078±1.257	2.145±1.050	0.840±1.003	1.252±0.902	0.661	0.000	0.000	0.002
Tyr/Leu	0.414±0.101	0.396±0.082	0.446±0.097	0.448±0.179	0.306	0.026	0.003	0.867
Val/Phe	2.802±5.564	2.436±1.607	2.480±4.711	4.238±2.695	0.519	0.005	0.002	0.000
C0	6.655±2.888	7.405±3.245	11.157±3.464	16.766±7.529	0.293	0.000	0.000	0.000
C2	2.893±2.130	2.722±1.721	2.290±2.556	2.615±1.974	0.593	0.330	0.740	0.239
C3	0.372±0.522	0.246±0.179	0.520±2.679	0.427±0.487	0.545	0.767	0.385	0.601
C4	0.330±0.581	0.244±0.500	3.583±26.968	0.259±0.335	0.966	0.968	0.994	0.057
C5	0.086±0.242	0.068±0.076	0.220±1.290	0.123±0.152	0.852	0.671	0.573	0.256
C6	0.297±0.479	0.234±0.374	3.111±22.175	0.241±0.352	0.970	0.970	0.996	0.046
C7	0.097±0.204	0.081±0.153	0.138±0.793	0.232±0.268	0.806	0.019	0.020	0.093
C8	0.098±0.105	0.086±0.073	0.085±0.152	0.298±0.302	0.672	0.000	0.000	0.000
C9	0.024±0.094	0.015±0.027	0.200±1.358	0.032±0.063	0.925	0.933	0.866	0.056
C10	0.115±0.108	0.110±0.077	0.121±0.239	0.200±0.210	0.830	0.000	0.001	0.001
C12	0.030±0.065	0.025±0.024	0.039±0.111	0.047±0.080	0.694	0.089	0.057	0.432
C14	0.018±0.025	0.027±0.039	0.029±0.111	0.036±0.076	0.389	0.048	0.370	0.426
C16	0.053±0.039	0.059±0.043	0.068±0.145	0.083±0.088	0.620	0.009	0.069	0.187
C17	0.018±0.095	0.011±0.023	0.010±0.008	0.024±0.057	0.374	0.441	0.116	0.051
C18	0.038±0.098	0.033±0.045	0.092±0.528	0.060±0.082	0.904	0.535	0.502	0.364
C20	0.015±0.035	0.011±0.031	0.115±0.932	0.027±0.074	0.948	0.849	0.815	0.147
C22	0.023±0.102	0.014±0.036	0.088±0.624	0.012±0.040	0.851	0.790	0.962	0.064
C24	0.024±0.049	0.025±0.065	0.065±0.466	0.035±0.068	0.969	0.736	0.794	0.330
C25	0.021±0.101	0.020±0.049	0.003±0.005	0.021±0.062	0.881	0.935	0.939	0.023
C26	0.021±0.098	0.018±0.033	0.022±0.148	0.018±0.045	0.806	0.799	0.985	0.715
C5:1	0.041±0.058	0.072±0.130	0.089±0.493	0.069±0.126	0.427	0.424	0.933	0.562
C8:1	0.294±0.195	0.288±0.121	0.225±0.206	0.579±0.322	0.843	0.000	0.000	0.000
C10:1	0.132±0.084	0.136±0.100	0.119±0.118	0.239±0.202	0.836	0.000	0.000	0.000
C10:2	0.044±0.091	0.038±0.051	0.037±0.083	0.069±0.114	0.636	0.026	0.015	0.003
C10:3	0.073±0.069	0.083±0.063	0.109±0.291	0.181±0.189	0.696	0.000	0.000	0.002
C12:1	0.090±0.128	0.075±0.049	0.066±0.109	0.130±0.114	0.343	0.003	0.000	0.000
C14:1	0.036±0.051	0.039±0.048	0.047±0.111	0.073±0.093	0.852	0.000	0.003	0.009
C14:2	0.030±0.066	0.025±0.027	0.059±0.245	0.073±0.091	0.811	0.016	0.018	0.403
C16:1	0.030±0.040	0.021±0.027	0.142±0.986	0.042±0.074	0.904	0.852	0.774	0.119
C18:1	0.070±0.106	0.067±0.062	0.166±0.852	0.109±0.090	0.956	0.499	0.512	0.310
C18:2	0.058±0.106	0.042±0.034	0.054±0.025	0.096±0.099	0.156	0.000	0.000	0.000
C20:1	0.011±0.027	0.013±0.033	0.035±0.294	0.020±0.049	0.912	0.645	0.765	0.456
C20:2	0.016±0.094	0.010±0.027	0.021±0.148	0.022±0.06	0.660	0.627	0.384	0.947
C20:3	0.035±0.283	0.015±0.024	0.022±0.148	0.022±0.056	0.378	0.528	0.748	0.994
C3DC	0.030±0.030	0.038±0.047	0.034±0.077	0.031±0.075	0.360	0.866	0.444	0.775
C4DC	0.045±0.111	0.037±0.042	0.053±0.232	0.067±0.124	0.738	0.231	0.162	0.426
C10OH	0.041±0.081	0.039±0.044	0.035±0.078	0.046±0.089	0.831	0.655	0.542	0.238
C6DC	0.016±0.023	0.018±0.026	0.022±0.112	0.028±0.062	0.849	0.160	0.290	0.468
C8DC	0.012±0.030	0.018±0.043	0.010±0.016	0.015±0.043	0.259	0.540	0.559	0.203
C10DC	0.047±0.044	0.049±0.047	0.053±0.146	0.060±0.075	0.856	0.242	0.391	0.536

C12DC	0.023±0.096	0.018±0.024	0.047±0.443	0.021±0.049	0.882	0.937	0.937	0.376
C14DC	0.015±0.047	0.021±0.050	0.005±0.006	0.113±0.166	0.622	0.000	0.000	0.000
C16DC	0.036±0.105	0.023±0.036	0.020±0.148	0.024±0.059	0.341	0.339	0.917	0.708
C18DC	0.029±0.105	0.019±0.037	0.008±0.006	0.015±0.046	0.235	0.061	0.631	0.333
C20DC	0.014±0.032	0.013±0.034	0.020±0.148	0.010±0.035	0.885	0.691	0.836	0.323
C4OH	0.043±0.066	0.073±0.137	0.094±0.491	0.032±0.072	0.434	0.761	0.293	0.062
C5OH	0.076±0.247	0.041±0.057	0.141±0.890	0.076±0.129	0.618	0.994	0.614	0.281
C6OH	0.040±0.075	0.033±0.051	0.068±0.447	0.058±0.119	0.841	0.578	0.487	0.734
C12OH	0.007±0.008	0.014±0.031	0.019±0.111	0.020±0.071	0.522	0.161	0.545	0.936
C14OH	0.009±0.008	0.014±0.025	0.020±0.111	0.014±0.040	0.634	0.531	0.936	0.494
C16:1OH	0.018±0.095	0.011±0.023	0.063±0.466	0.024±0.057	0.839	0.866	0.724	0.206
C16OH	0.009±0.009	0.009±0.009	0.020±0.148	0.011±0.035	0.966	0.793	0.849	0.351
C18:1OH	0.019±0.097	0.007±0.008	0.061±0.466	0.018±0.056	0.745	0.984	0.758	0.170
C18OH	0.016±0.094	0.009±0.027	0.033±0.294	0.019±0.058	0.784	0.865	0.671	0.491
C20OH	0.022±0.100	0.022±0.049	0.113±0.932	0.019±0.057	0.993	0.968	0.964	0.123
C0/C2	3.249±2.959	3.234±2.470	8.551±6.836	5.174±4.358	0.987	0.018	0.035	0.000
C0/C16	125.77±70.048	136.069±77.635	211.195±84.230	155.937±143.546	0.479	0.020	0.173	0.000
C3/C0	0.063±0.091	0.039±0.032	0.039±0.150	0.030±0.036	0.075	0.006	0.521	0.458
C3/C2	0.171±0.214	0.115±0.107	0.233±0.665	0.115±0.186	0.301	0.247	0.994	0.012
C3/C16	5.659±4.121	4.530±3.267	4.781±2.461	3.202±3.607	0.040	0.000	0.016	0.001
C4/C3	1.006±1.775	0.884±1.638	2.071±3.242	0.561±0.845	0.696	0.107	0.297	0.000
C5/C4	0.507±0.487	0.496±0.548	0.652±0.474	0.267±0.338	0.872	0.000	0.001	0.000
C5DC/C8	0.468±0.392	0.529±0.510	0.433±0.272	0.229±0.325	0.264	0.000	0.000	0.000
C5DC/C16	0.718±0.568	0.761±0.735	0.511±0.402	0.542±0.713	0.638	0.027	0.015	0.691
C8/C2	0.038±0.044	0.033±0.029	0.049±0.049	0.075±0.102	0.681	0.000	0.000	0.003
C8/C3	0.320±0.323	0.341±0.334	0.311±0.220	0.433±0.529	0.725	0.034	0.126	0.019
C8/C1	0.835±0.535	0.775±0.568	0.835±0.321	0.578±0.537	0.404	0.000	0.006	0.000
C8/C12	3.052±2.232	3.130±3.057	2.690±1.546	2.212±2.525	0.820	0.006	0.007	0.105
C8/C16	1.562±1.126	1.602±1.372	1.286±0.775	1.752±1.654	0.837	0.272	0.441	0.006
C14:1/C16	0.559±0.424	0.594±0.399	0.666±0.346	0.550±0.548	0.582	0.885	0.497	0.036
C16OH/C16	0.192±0.200	0.212±0.279	0.137±0.140	0.100±0.191	0.508	0.000	0.000	0.149
C24/C22	0.564±0.685	0.584±0.731	1.163±0.978	0.339±0.655	0.866	0.037	0.044	0.000
C25/C22	0.208±0.389	0.265±0.433	0.270±0.444	0.094±0.289	0.346	0.031	0.004	0.001
C26/C2	0.429±0.703	0.486±0.736	0.817±0.656	0.311±0.637	0.573	0.197	0.088	0.000
C26/C22	0.369±0.562	0.504±0.653	0.817±0.652	0.254±0.622	0.152	0.173	0.008	0.000
C26/C24	0.463±0.598	0.630±0.672	0.883±0.699	0.382±0.654	0.092	0.360	0.013	0.000

Supplementary Table S2. HPLC Condition for 21 AAs and 55 Carnitines Detection

Time(min)	Flow Rate(μL/min)	Acetonitrile (%)
0.1	150	100
1	60	100
1.1	500	100
2	500	100
2.1	150	100

Supplementary Table S3. Optimized Parameters for the Detection of 21 AAs and 55 Carnitines by Mass Spectrometry on Q-trap

Parameters	Value
CUR	20 psi
CAD	Medium
IS	5500
TEM	450℃
GS1	40 psi
GS2	50 psi
DP	35
EP	7
CE	22
CXP	3

Supplementary Table S4. Logistic Regression Analysis of Predictive Factors for NPWI

			95% CI	
**Analytes**	***P***	**OR**	**Lower Limit**	**Upper Limit**
C8:1	0.010	50.610	2.601	984.879
AMH	0.001	1.522	1.191	1.945
Pip	0.449	1.011	0.983	1.040
Gln	0.803	1.010	0.932	1.096
C4/C3	0.025	0.687	0.495	0.954
Gln/Cit	0.783	0.939	0.600	1.469
Arg/Orn	0.000	128.985	12.963	1283.447
